# HGF-MET Signaling Shifts M1 Macrophages Toward an M2-Like Phenotype Through PI3K-Mediated Induction of Arginase-1 Expression

**DOI:** 10.3389/fimmu.2020.02135

**Published:** 2020-09-02

**Authors:** Nao Nishikoba, Kotaro Kumagai, Shuji Kanmura, Yuko Nakamura, Mayumi Ono, Hiromi Eguchi, Tomomi Kamibayashiyama, Kohei Oda, Seiichi Mawatari, Shiroh Tanoue, Shinichi Hashimoto, Hirohito Tsubouchi, Akio Ido

**Affiliations:** ^1^Digestive and Lifestyle Diseases, Department of Human and Environmental Sciences, Kagoshima University Graduate School of Medical and Dental Sciences, Kagoshima, Japan; ^2^Department of Gastroenterology and Hepatology, Kagoshima City Hospital, Kagoshima, Japan

**Keywords:** HGF-MET signaling, macrophage, phenotypic alteration, arginase-1, PI3K pathway

## Abstract

**Backgrounds and Aims:** Hepatocyte Growth Factor (HGF)-MET signaling is known to promote biological functions such as cell survival, cell motility, and cell proliferation. However, it is unknown if HGF-MET alters the macrophage phenotype. In this study, we aimed to study the effects of HGF-MET signaling on the M1 macrophage phenotype.

**Methods and Materials:** Bone marrow-derived macrophages (BMDMs) isolated from mice were either polarized to an M1 phenotype by IFN-γ and LPS treatment or to an M2 phenotype by IL-4 treatment. Changes in M1 or M2 markers induced by HGF-MET signaling were evaluated. Mechanisms responsible for alternations in the macrophage phenotype and intracellular metabolism were analyzed.

**Results:** c-Met was expressed especially in M1 macrophages polarized by treatment with IFN-γ and LPS. In M1 macrophages, HGF-MET signaling induced the expression of *Arg-1* mRNA and secretion of IL-10 and TGF-β1 and downregulated the mRNA expression of *iNOS, TNF-*α, and *IL-6*. In addition, activation of the PI3K pathway and inactivation of NFκB were also observed in M1 macrophages treated with HGF. The increased Arg-1 expression and IL-10 secretion were abrogated by PI3K inhibition, whereas, no changes were observed in TNF-α and IL-6 expression. The inactivation of NFκB was found to be independent of the PI3K pathway. HGF-MET signaling shifted the M1 macrophages to an M2-like phenotype, mainly through PI3K-mediated induction of Arg-1 expression. Finally, HGF-MET signaling also shifted the M1 macrophage intracellular metabolism toward an M2 phenotype, especially with respect to fatty acid metabolism.

**Conclusion:** Our results suggested that HGF treatment not only promotes regeneration in epithelial cells, but also leads to tissue repair by altering M1 macrophages to an M2-like phenotype.

## Introduction

Hepatocyte Growth Factor (HGF) from blood plasma of patients with fulminant hepatic failure has been discovered to be a factor that strongly stimulates DNA synthesis in hepatocytes (1). Pro-HGF is secreted by mesenchymal cells in a single-chain, biologically inactive form and is cleaved to its bioactive form by extracellular proteases. Mature HGF consists of an α- and β-chain linked by a disulfide bond (2). *In vivo*, increased HGF expression is observed after experimental hepatic, renal, cardiac, or pulmonary injury and is associated with tissue repair (3). The ligand HGF binds to the tyrosine-protein kinase Met (c-MET), a single-pass transmembrane, disulfide-linked α/β heterodimer receptor (4). c-MET is expressed in epithelial cells of the liver, bone marrow, pancreas, kidneys, and muscles (5). HGF-MET signaling is known to promote angiogenesis, cellular motility, growth, invasion, morphological differentiation, embryological development, tissue regeneration, and wound healing in various organs (6). Based on these data, we attempted to use HGF in clinical treatment and confirmed its safety for acute liver failure patients in a coma (7). However, further work on HGF-MET signaling is necessary in several areas to realize the clinical potential of HGF. One of these areas is its impact on immune cells.

Macrophages are innate immune cells involved in homeostasis, the immune response, inflammation, regeneration, and the resolution of inflammation in tissues (8). We have also previously shown *in vivo* that macrophages that infiltrate into an injured liver during the recovery phase are crucial for the attenuation of inflammation and tissue repair (9). Macrophage polarization states are mainly divided into two types, the pro-inflammatory or classically activated M1 phenotype and the anti-inflammatory or alternatively activated M2 phenotype (10). LPS and/or IFN-γ can induce polarization to M1 macrophages that are capable of pro-inflammatory responses and produce pro-inflammatory molecules such as TNF-α and IL-6 (11). IL-4, IL-13, or IL-10 can induce polarization to M2 macrophages that are capable of anti-inflammatory responses and tissue repair (12). The functions and phenotypes of macrophages are diverse and vary with different microenvironments. Macrophages dynamically alter their phenotype and switch from an M1 to M2 phenotype during different phases (13). However, little is known about the factors that promote conversion from a pro-inflammatory phenotype (M1) to an anti-inflammatory and restorative phenotype (M2) during tissue repair.

Previous reports have shown that c-Met is expressed not only by epithelial cells but also by macrophages, monocytes, dendritic cells, and T cells and that in these cells, HGF-MET signaling has an effect on cellular responses (14–20). Therefore, we hypothesized that HGF-MET signaling is involved in phenotypic changes in macrophages. In this study, we demonstrated a mechanism underlying an HGF-MET signaling-mediated shift from M1 to M2-like macrophages through the phosphatidylinositol-3 kinase (PI3K)-dependent induction of Arg-1 expression. Collectively, our results suggest that HGF treatment might not only promote regeneration in epithelial cells but also shift M1 macrophages to an M2-like phenotype, leading to tissue repair.

## Materials and Methods

### Mice

Specific pathogen-free, C57BL/6J mice were sourced from KBT Oriental Co., Ltd. (Tosu, Japan). All animal experimental procedures were performed in accordance with the protocols and guidelines for animal experiments that were approved by the institutional animal care and use committees of Kagoshima University (Permit Numbers: MD19057). All animals were housed with standard chow^1^ and tap water under standard conditions, in a 24°C temperature-controlled environment with a 12-h light/12-h dark cycle. Mice were monitored daily, and were euthanized by cervical dislocation under anesthesia if they showed distress during experiments. All efforts were made to minimize animal suffering.

### Isolation of Bone Marrow Cells and Cell Culture

Bone marrow-derived macrophages (BMDM) were obtained from the femurs and tibias of sacrificed mice (8–10 weeks of age) by flushing with Dulbecco's Modified Eagle Medium (DMEM; Thermo Fisher Scientific, MA, USA) containing 10% fetal bovine serum (FBS; Thermo Fisher Scientific, MA, USA) and 1% Penicillin-Streptomycin (P/S; Thermo Fisher Scientific, MA, USA). Cells were filtered, washed, re-suspended, and cultured in differentiation medium, containing DMEM with 10% FCS, 1% P/S, and 25 ng/mL macrophage colony-stimulating factor (M-CSF; R & D systems, MN, USA). Cells were cultured in differentiation medium for 7 days, following which, M-CSF was depleted. These cells were defined as M0 macrophages (> 95% of population F4/80-positive). For macrophage polarization, cells were seeded in 12-well plates (1 × 10^6^ cells/well), and were treated for 48 h either with 20 ng/mL IFN-γ (PeproTech, NJ, USA) and 10 ng/mL LPS (Sigma-Aldrich, MO, USA) in media (DMEM with 10% FCS, 1% P/S) to induce an M1 phenotype or with 20 ng/mL IL-4 (PeproTech, NJ, USA) to induce an M2 phenotype. Cells were treated for 24 h with recombinant human HGF (E3112; EA Pharma Co., Ltd, Tokyo, Japan) 24 h after polarization to an M1 or M2 phenotype.

### Cell Culture and Treatment With PI3K Inhibitor

After macrophages were polarized to an M1 phenotype, they were cultured in media with or without HGF treatment for 24 h. Cells were also treated for 1 or 24 h either with 20 μM of the PI3K inhibitor LY294002 (Selleck chemicals, Houston, USA) or the treatment vehicle dimethyl sulfoxide (DMSO, Nacalai Tesque Inc., Kyoto, Japan), before being harvested. This inhibitor dose was previously reported to be effective (21).

### RNA Isolation and RT-qPCR

Total RNA was extracted from cells using the TRIzol reagent (Thermo Fisher Scientific, MA, USA). RNA purity was confirmed by spectrophotometry, and A_260_/A_280_ ratios ranged from 1.9 to 2.1. First-strand cDNA was synthesized from 500 ng of the total RNA using a PrimeScript RT Master Mix (Takara Bio Inc., Shiga, Japan). Real-time PCR was performed using TB Green Premix Ex Taq II (Takara Bio Inc., Shiga, Japan) and the ABI Prism 7700 sequence detection system (Applied Biosystems, Foster City, CA, USA). Data were collected and analyzed using the Step One Plus Real-Time PCR System (Applied Biosystems, Foster City, CA, USA). After calculation of the cycle threshold (Ct) value for each sample, relative levels were calculated and normalized to β-actin. PCR conditions were as follows: initial holding period at 95°C for 30 s, followed by 40 cycles of a 2-step program consisting of denaturation at 95°C for 5 s and annealing, and polymerization at 60°C for 34 s. All reactions were performed in duplicate. The following mouse primers were used (f, forward; r, reverse; Takara Bio Inc. Shiga, Japan): β-actin, iNOS, CD86, TNF-α, IL-6, Arg-1, CD206, IL-10, TGF-β1, c-Met, Slc2a1, hexokinase, pyruvate kinase, Slc1a5, glutaminase, glutamate dehydrogenase, CD36, fatty acid synthase, and acyl-CoA synthase (summarized in Supplemental Table 1A). Gene expression values were calculated using the comparative ΔΔCt method and the Ct values were normalized to those of β-actin and are expressed relative to mean level in M0 macrophages not treated with HGF, which is arbitrarily defined as 1.

### Western Blotting

Cells were lysed in RIPA buffer (Sigma Aldrich, MO, USA) with both a protease inhibitor (Nacalai Tesque Inc., Kyoto, Japan) and a phosphatase inhibitor (Nacalai Tesque Inc., Kyoto, Japan). The same amount of cell lysate sample (10 μL) was used and separated using 4–20% SDS polyacrylamide gel electrophoresis before being electrophoretically transferred to a polyvinylidene fluoride membrane (Bio-Rad Laboratories, Inc., CA, USA). The membranes were blocked for 1 h at room temperature using the SuperBlock™ (PBS) Blocking Buffer (Thermo Fisher Scientific, MA, USA) and were probed by incubating them overnight at 4°C with respective primary antibodies diluted in Solution 1 of Can Get Signal Immunoreaction Enhancer Solution (TOYOBO Co., Ltd., Osaka, Japan). The membranes were then incubated with horseradish peroxidase (HRP)-conjugated anti-mouse antibodies (Cell Signaling Technology, MA, USA). The protein bands were visualized with an enhanced chemiluminescence system (GE Healthcare, IL, USA) and a Fusion Solo S6 (Vilber Lourmat, France). Western blot bands showing phosphorylation of intracellular signaling were quantified by densitometry with Image J software (National Institutes of Health, MD, USA). All antibodies used in this study are listed in Supplemental Table 1B.

### ELISA

The concentration of TNF-α, IL-6, IL-10, and TGF-β1 in cell-free supernatants was measured using Mouse Quantikine TNF-α, IL-6, IL-10, and TGF-β1 ELISA Kits, respectively (R & D systems, MN, USA).

### Statistical Analysis

All data are expressed as the mean with a scatterplot showing individual data points. Statistical analysis was performed using either an unpaired Student's *t*-test or a one-way ANOVA with appropriate *post-hoc* tests (Graph Pad Prism version 8.4.0). *P* < 0.05 were considered statistically significant. *In vitro* experiments were independently performed two or more times.

## Results

### Expression and Phosphorylation of c-Met Is Induced Especially in M1 Macrophages

**Figure 1:**
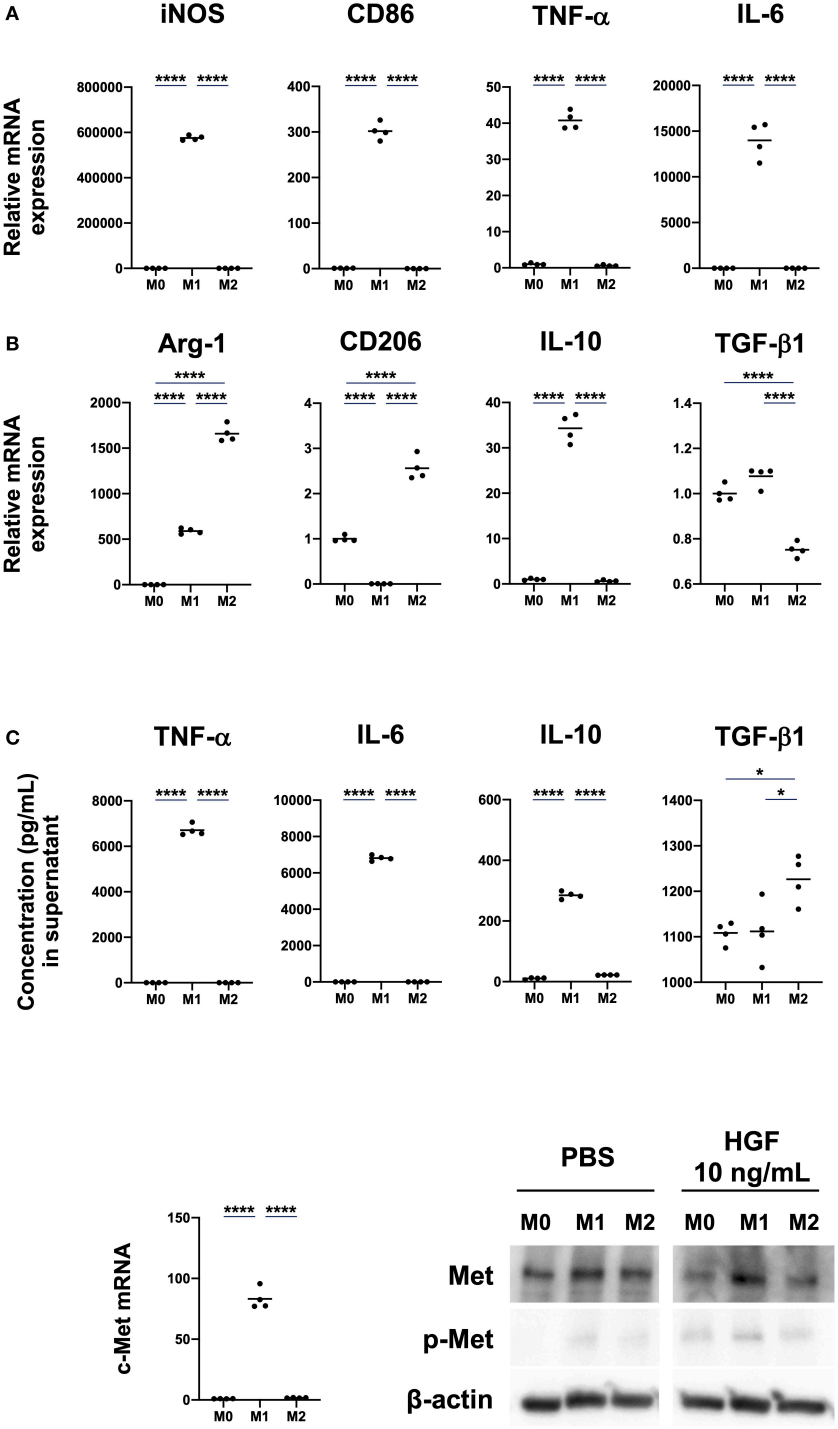
M1 macrophages induced by IFN-γ and LPS treatment show enhanced expression and phosphorylation of c-Met. **(A)** Expression of M1 and M2 markers. Bone marrow-derived macrophages (BMDMs; M0 macrophages) were differentiated by treatment of bone-marrow cells with M-CSF (25 ng/mL) for 7 days. Macrophages were polarized to M1 and M2 phenotypes by treatment for 48 h with IFN-γ and LPS or IL-4, respectively. Total RNA extraction and RT-qPCR were used to analyze mRNA expression of the M1 markers *iNOS, CD86, TNF-*α, and *IL-6* and M2 markers *Arg-1, CD206, IL-10*, and *TGF-*β*1*. **(B)** Concentrations of cytokines in cell culture supernatant. ELISA was used to measure spontaneous secretion of TNF-α, IL-6, IL-10, and TGF-β1 in cell culture supernatant from M0, M1, and M2 macrophages. **(C)** Expression of c-Met in M0, M1, and M2 macrophages. c-Met expression was compared using RT-qPCR and western blot. The RT-qPCR Ct values were normalized to those of β-actin and have been expressed relative to mean level in M0, which is arbitrarily defined as 1. β-actin was used as a loading control for western blots. The results are represented as the mean and a scatter plot of individual data points (*n* = 4). One-way ANOVA, *P < 0.05 and ****P < 0.0001.

We first investigated if M0, M1, and M2 macrophages expressed the c-Met. M0 macrophages were induced by treating bone marrow cells with M-CSF for 7 days. Macrophages were then polarized to either an M1 phenotype, by treating with IFN-γ and LPS, or an M2 phenotype via treatment with IL-4. M1 macrophages significantly upregulated mRNA expression of the M1 markers *iNOS, CD86, TNF-*α, and *IL-6*. mRNA expression of the M2 markers *Arg-1* and *CD206* was significantly upregulated in M2 macrophages, but mRNA expression of *IL-10* was significantly upregulated in M1 macrophages (Figure 1A). Further, though the spontaneous secretion of TNF-α, IL-6, and IL-10 was significantly increased in the cell culture supernatant of M1 macrophages, TGF-β1 secretion was significantly increased in M2 macrophages (Figure 1B). c-Met expression was significantly upregulated in M1 macrophages. We also found that HGF treatment induced the phosphorylation of c-Met, especially in M1 macrophages (Figure 1C).

### HGF-MET Signaling Shifts M1 Macrophages to an M2-Like Phenotype

**Figure 2:**
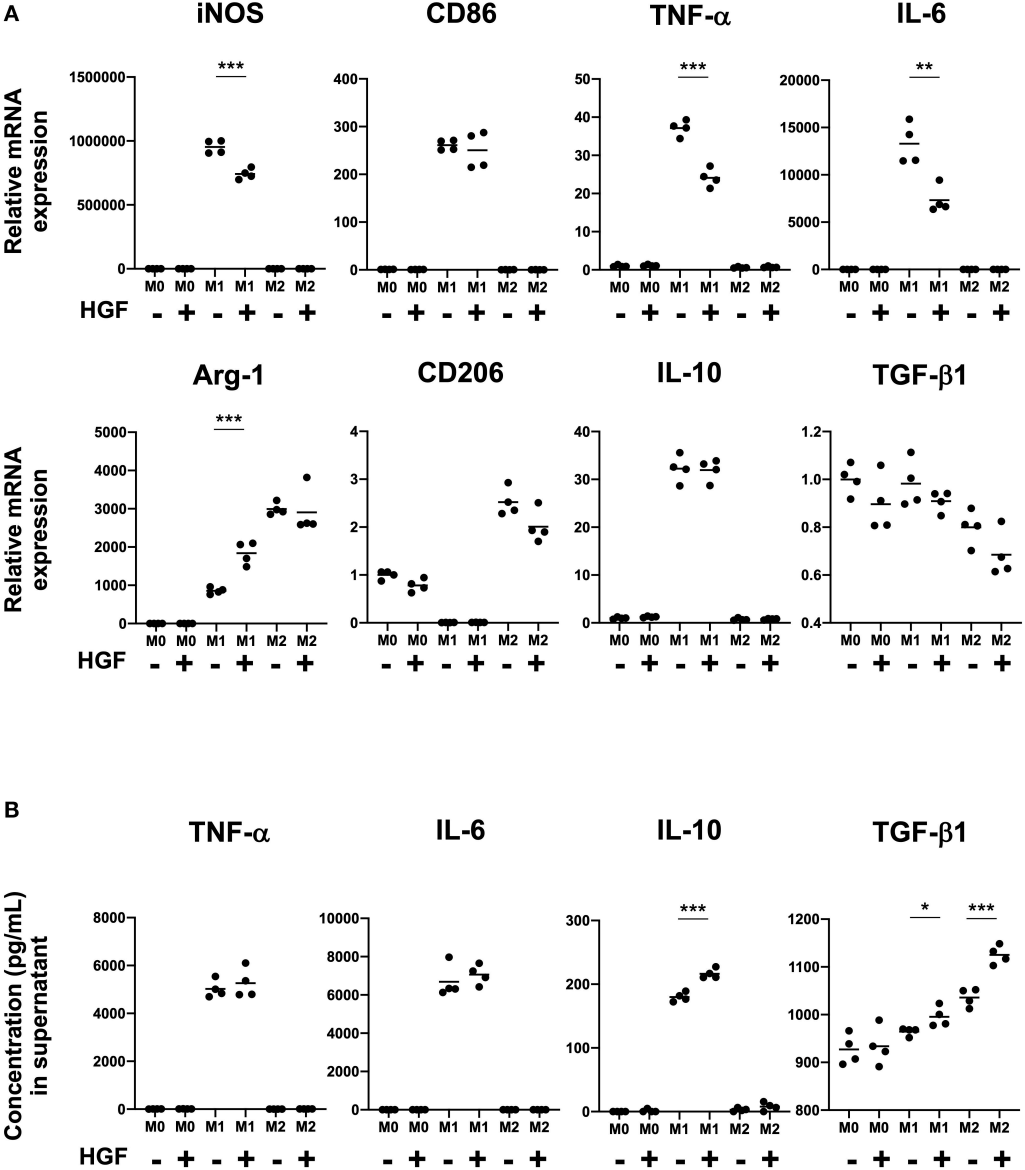
HGF-MET signaling shifts M1 macrophages to an M2-like phenotype. **(A)** Effect of HGF treatment on expression of M1 and M2 markers. After differentiation to an M0 phenotype, macrophages were polarized to an M1 phenotype by treatment for 24 h with IFN-γ and LPS and an M2 phenotype by treatment with IL-4. M1 and M2 macrophages were then cultured for 24 h with either PBS or HGF (10 ng/mL). RT-qPCR was used to analyze mRNA expression of the M1 markers *iNOS, CD86, TNF-*α, and *IL-6* and M2 markers *Arg-1, CD206, IL-10*, and *TGF-*β*1*. Ct values were normalized to those of β*-actin* and are expressed relative to mean level in M0 macrophages not treated with HGF, which was arbitrarily defined as 1. **(B)** Effect of HGF treatment on secretion of cytokines by M1 and M2 macrophages. ELISA was used to measure the secretion of TNF-α, IL-6, IL-10, and TGF-β*1* in cell culture supernatants from M0, M1, and M2 macrophages that were treated or not treated with HGF. The results are represented as the mean and a scatter plot showing individual data points (*n* = 4). Unpaired Student's *t*-test (HGF– vs. HGF+), *P < 0.05, **P < 0.01, and ***P < 0.001.

We next investigated if HGF-MET signaling was involved in determination of the macrophage phenotype. We had previously shown that the mRNA expression of *iNOS, CD86, TNF-*α, and *IL-6* was upregulated in M1 macrophages and that *Arg-1* and *CD206* mRNA expression was upregulated in M2 macrophages. For this study, we focused on the phenotypic changes in macrophages with or without HGF treatment. There was no significant difference observed between M0 and M2 macrophages regardless of HGF treatment. However, for M1 macrophages, we observed significant downregulation of the mRNA expression of M1 markers, including *iNOS, TNF-*α, and *IL-6*, and the upregulation of *Arg-1* with HGF treatment compared to levels in untreated cells (Figure 2A). Further, though HGF treatment significantly increased the secretion of IL-10 and TGF-β1 in the cell culture supernatant of M1 macrophages, no difference was observed for TNF-α and IL-6. TGF-β1 secretion was also significantly increased in both M1 and M2 macrophages that were treated with HGF (Figure 2B).

### HGF-MET Signaling Induces Arg-1 Expression Through the PI3K-Dependent Phosphorylation of CREB and C/EBPβ

**Figure 3:**
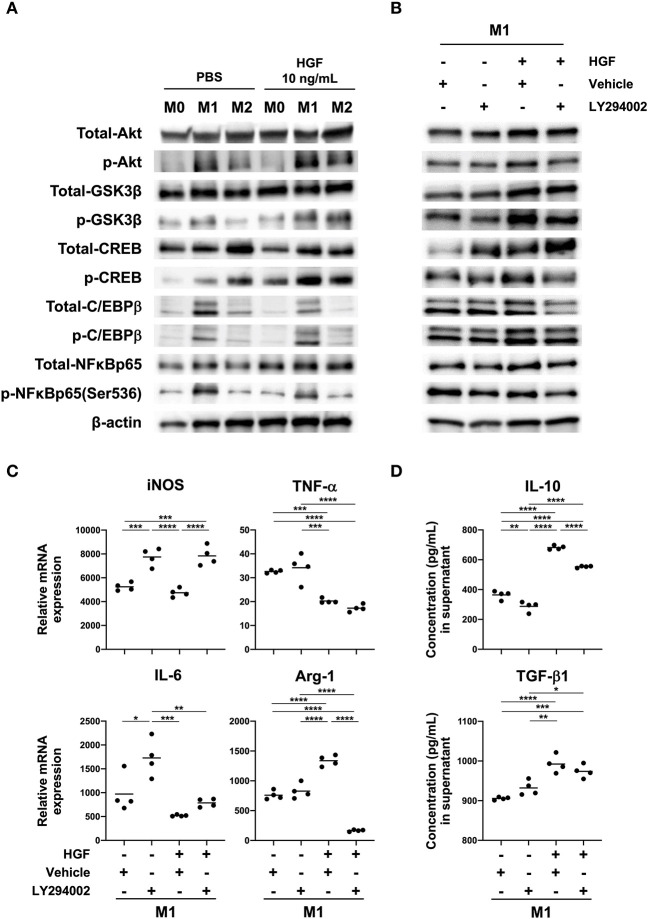
HGF-MET signaling induces PI3K-mediated Arg-1 expression. **(A)** Effect of HGF treatment on activity of the PI3K pathway in macrophages. Bone marrow-derived macrophages (BMDMs; M0 macrophages) were induced through the treatment of bone-marrow cells with M-CSF (25 ng/mL) for 7 days. Macrophages were polarized to M1 and M2 phenotypes with treatment for 48 h using IFN-γ and LPS or IL-4, respectively. Western blotting was used to analyze the levels of total and phosphorylated Akt, GSK-3β, CREB, C/EBPβ, and NF-κB. **(B)** Effects of HGF treatment on downstream PI3K signaling in the presence and absence of PI3K inhibitor. M1 macrophages were treated with a PI3K inhibitor (LY294002) for 1 h after PBS or HGF treatment for 24 h. Western blotting was used to analyze the levels of total and phosphorylated Akt, GSK-3β, CREB, C/EBPβ, and NF-κB. β-actin was used as a loading control. **(C)** Effect of PI3K inhibitor treatment on the expression of M1 and M2 markers with or without HGF treatment. RT-qPCR was used to compare mRNA expression of *iNOS, TNF-*α, *IL-6*, and *Arg-1* in M1 macrophages that were treated with or without PI3K inhibitor and HGF for 24 h. The Ct values were normalized to those of β-actin and are expressed relative to mean levels in M0 macrophages not treated with HGF, which is arbitrarily defined as 1. **(D)** ELISA was used to measure the secretion of IL-10 and TGF-β*1*. Data are represented as the mean and a scatterplot showing individual data points (*n* = 4). One-way ANOVA, *P < 0.05, **P < 0.01, ***P < 0.001, and ****P < 0.0001.

To identify mechanisms underlying changes in the M1 macrophage phenotype, we examined PI3K activity. We found that HGF treatment induced the phosphorylation of Akt, GSK3β, CREB, and C/EBPβ in M1 macrophages compared to that in untreated cells. We also observed the inactivation of NF-κB (Figure 3A and Supplemental Figure 1A). A PI3K inhibitor, LY294002, was used to verify that the PI3K pathway was crucial for changes in the M1 phenotype. Combinatorial treatment of M1 macrophages with HGF and LY294002 inhibited the phosphorylation of Akt, GSK3β, CREB, and C/EBPβ and enhanced the inactivation of NF-κB compared to that in cells treated with HGF and DMSO (Figure 3B and Supplemental Figures 1B,C). Differences between M1 macrophages that were treated with DMSO and HGF and the cells treated only with DMSO were similar to that previously observed (Figure 2A), with the exception of *iNOS* and *IL-6* mRNA expression (Figure 3C). It is known that DMSO treatment on its own also downregulates the expression of *iNOS* and *IL-6* (22). Therefore, there was a trend toward a decreased expression of *iNOS* and *IL-6*, but with no significant difference. In M1 macrophages treated with HGF and LY294002, *Arg-1* expression was downregulated, *iNOS* expression was significantly upregulated, and no change was observed in *TNF-*α and *IL-6* compared to those with HGF and DMSO treatment (Figure 3C). Combinatorial treatment of M1 macrophages with HGF and LY294002 also significantly decreased the secretion of IL-10 but not TGF-β1 when compared to that in cells treated with HGF and DMSO (Figure 3D).

### HGF-MET Signaling Shifts M1 Macrophage Intracellular Metabolism Toward an M2-Like Phenotype

**Figure 4:**
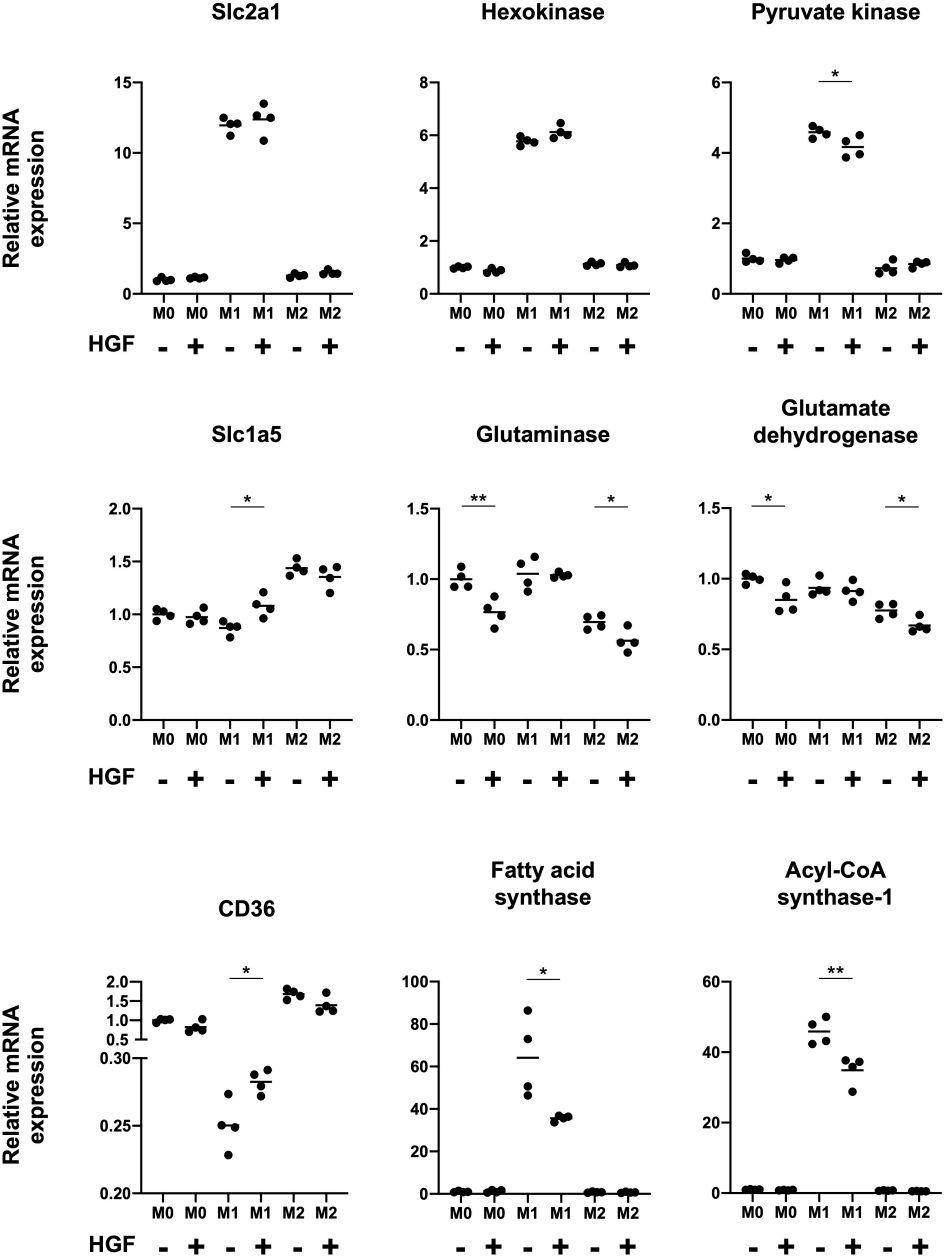
HGF-MET signaling shifts M1 macrophage intracellular metabolism to an M2-like phenotype. Effect of HGF treatment on macrophage intracellular metabolism. RT-qPCR was used to analyze mRNA expression of genes involved in glucose metabolism (*Slc2a1*, hexokinase, and pyruvate kinase), amino acid metabolism (*Slc1a5*, glutaminase, and glutamate dehydrogenase), and fatty acid metabolism (*CD36*, fatty acid synthase, and acyl-CoA synthase-1). The Ct values were normalized to those of β-actin and have been expressed relative to mean level in M0 macrophages not treated with HGF, which is arbitrarily defined as 1. The data are represented as the mean and a scatterplot showing individual data points (*n* = 4). Unpaired Student's *t*-test, *P < 0.05 and **P < 0.01.

Finally, we studied changes in M1 macrophage intracellular metabolism when treated with HGF to determine if there was a shift toward an M2-like phenotype. We evaluated the mRNA expression of transporters and enzymes involved in glucose, amino acid, and fatty acid metabolism in M1 macrophages. The data for M0 and M2 macrophages was shown as a reference. With HGF treatment, we found that pyruvate kinase, which is involved in glucose metabolism, was significantly downregulated, and *Scl1a5*, which is involved in amino acid metabolism, was significantly upregulated. With regards to fatty acid metabolism, M1 macrophages treated with HGF significantly upregulated *CD36* expression and significantly downregulated fatty acid synthase and acyl-CoA synthase-1 (Figure 4).

## Discussion

In this study, we found that c-Met was expressed especially by M1 macrophages that were treated with IFN-γ and LPS. We also found that HGF-MET signaling shifted M1 macrophages toward an M2-like phenotype through the PI3K-dependent induction of Arg-1 expression. The factors and mechanisms that promote a transition from a pro-inflammatory to an anti-inflammatory phenotype remain unknown. In this report, we have identified a mechanism of HGF-MET signaling that alters the macrophage phenotype, which is potentially one factor that addresses this question.

Macrophages were polarized to either an M1 phenotype by IFN-γ and LPS treatment or an M2 phenotype by IL-4 treatment. To characterize the macrophages, expression of the M1 markers iNOS, CD86, TNF-α, and IL-6 and the M2 markers Arg-1, CD206, IL-10, and TGF-β were evaluated (23). Our data showed that all M1 markers were exclusively expressed by M1 macrophages and that M2 markers were expressed by both M2 and M1 macrophages. In particular, IL-10 was expressed more strongly by M1 than by M2 macrophages. This was consistent with a previous report that showed M1 macrophages that are polarized by IFN-γ and LPS treatment express and secrete IL-10 (24). Further, it has also been reported that macrophages derived from both human peripheral blood mononuclear cells (PBMCs) and BMDMs that are polarized to an M1 phenotype express and secrete higher levels of IL-10 compared than M2 macrophages polarized by IL-4 treatment (25, 26).

Previous reports have shown that human monocytes isolated from PBMCs and cord blood cells express c-Met (16, 17). It has also been suggested that HGF-MET signaling affects the differentiation and function of monocytes and macrophages (14, 15). However, whether HGF-MET signaling affects differentiated macrophages remains to be elucidated. We also showed high levels of c-Met expression and phosphorylation in M1 macrophages treated with HGF and that HGF-MET signaling shifts M1 macrophages toward an M2-like phenotype.

It is known that PI3K is a major pathway activated downstream of HGF-MET signaling (5, 27). Phosphorylated c-Met activates PI3K, promoting cell viability, motility, cell morphogenesis, and cell survival (28, 29). However, whether HGF-MET and downstream PI3K-mediated signaling alter macrophage phenotypes remains unknown. Western blotting confirmed that HGF-MET signaling activated the PI3K pathway, and we observed phosphorylation of Akt, GSK3β, CREB, and C/EBPβ, with the deactivation of NF-κB. These changes induced by HGF treatment were abrogated by co-treatment with a PI3K inhibitor, with the exception of NF-κB, which remained deactivated regardless of PI3K inhibition. This suggests that during HGF-MET signaling, the phosphorylation of GSK3β, CREB, and C/EBPβ is regulated by PI3K but that the deactivation of NF-κB is regulated by an alternative pathway. We also noted that whereas the upregulation of *Arg-1* mRNA expression was abrogated by co-treatment with a PI3K inhibitor, the inhibitor had no effect on the downregulation of *TNF-*α and *IL-6* mRNA expression. iNOS expression was upregulated with the inhibition of PI3K regardless of HGF treatment, indicating that the upregulation of iNOS takes place independent of HGF-MET signaling. These data also suggest that PI3K signaling induced by LPS treatment might downregulate iNOS expression (30). Collectively, these results indicate that the induction of Arg-1 expression by HGF-MET signaling is PI3K-dependent and that the downregulation of *TNF-*α and *IL-6* mRNA expression occurs independently of PI3K.

Recent studies have demonstrated that there is a relationship between macrophage phenotypes and their intracellular metabolism (31, 32). M1 macrophages are mainly dependent on glycolysis for metabolism, whereas M2 macrophages are dependent on oxidative forms of metabolism. As such, M1 macrophages have increased glycolysis, activate the pentose-phosphate pathway, and fatty acid synthesis. In contrast, M2 macrophages display enhanced OXPHOS metabolism, fatty acid oxidation, and amino acid uptake (33, 34). We observed upregulation of the amino acid transporter *Slc1a5* and the fatty acid transporter *CD36* in M1 macrophages treated with HGF. Moreover, the downregulation of pyruvate kinase, fatty acid synthase, and acyl-CoA synthase-1 was shown, suggesting that they are related to the M2 phenotype. These results indicate that HGF-MET signaling not only alters the phenotype of M1 macrophages but also their intracellular metabolism.

For this study, we utilized a mouse BMDM model and not macrophages derived from PBMCs. Experiments that showed a relationship between HGF-MET signaling and an altered macrophage phenotype were also not conducted *in vivo* but rather *in vitro*, with macrophages that were polarized by IFN-γ and LPS or IL-4. However, the primary aim of this study was to study the effect of HGF-MET signaling on macrophage phenotypes. This was previously unknown and we have obtained new insight by providing confirmation that HGF-MET signaling alters the M1 macrophage phenotype in an *in vitro* BMDM model. Further work is necessary to verify that HGF-MET signaling also alters the phenotype of human macrophages from PBMCs *in vitro* and in *in vivo* mouse models. This is key to our future aim of the clinical application of HGF as a therapeutic.

In conclusion, we report a mechanism of HGF-MET signaling that shifts M1 macrophages toward an M2-like phenotype. Our data identifies a possible factor involved in changes from a pro-inflammatory to an anti-inflammatory phenotype during tissue repair. Furthermore, our results suggest that HGF treatment might not only promote regeneration in epithelial cells but could also lead to tissue repair by shifting M1 macrophages toward an M2-like phenotype.

## Data Availability Statement

All datasets generated for this study are included in the article/Supplementary Material.

## Ethics Statement

The animal study was reviewed and approved by Institutional animal care and use committees of Kagoshima University.

## Author Contributions

NN designed the study, performed the experiments, analyzed the data, and wrote the manuscript. YN, MO, HE, and TK performed the experiments. SK, KO, SM, ST, SH, HT, and AI supervised the experiments and reviewed the manuscript. KK designed the study and wrote the manuscript. All authors contributed to the article and approved the submitted version.

## Conflict of Interest

AI has received honoraria from Eisai Co., Ltd. The remaining authors declare that the research was conducted in the absence of any commercial or financial relationships that could be construed as a potential conflict of interest.

## Footnotes

^1^We used standard chow from CLEA Japan, Inc. Product name is CE-2. This diet is a GLP-compliant, standard rodent diet consisting mainly of vegetable protein (soybean meal) with a proper balance of animal protein.

## Supplementary Material

Supplemental Figure 1 **(A,B)** Quantification of Western blot bands based on Figures 3A,B. M1 macrophages were treated with a PI3K inhibitor (LY294002) for 1 h after PBS or HGF treatment for 24 h. **(C)** Effects of HGF treatment on phosphorylation of C/EBPβ in the presence and absence of a PI3K inhibitor. M1 macrophages were treated with a PI3K inhibitor (LY294002) with or without HGF for 24 h. Western blot bands were quantified by densitometry. The values were normalized to those of β-actin. The data are represented as the mean and a scatterplot showing individual data points (*n* = 4). Unpaired Student's *t*-test for **(A)**, ^**^*P* < 0.01, ^***^*P* < 0.001, and ^****^*P* < 0.0001. One-way ANOVA for **(B)**, ^**^*P* < 0.01, ^***^*P* < 0.001, and ^****^*P* < 0.0001.

Supplemental Table 1 All primers and antibodies used in this study are listed.
