# Supplementary figures and images for: HGF-MET Signaling Shifts M1 Macrophages Toward an M2-Like Phenotype Through PI3K-Mediated Induction of Arginase-1 Expression
Source: Front Immunol. 2020 Sep 2;11:2135. doi: 10.3389/fimmu.2020.02135 (PMC7492554; doi:10.3389/fimmu.2020.02135)

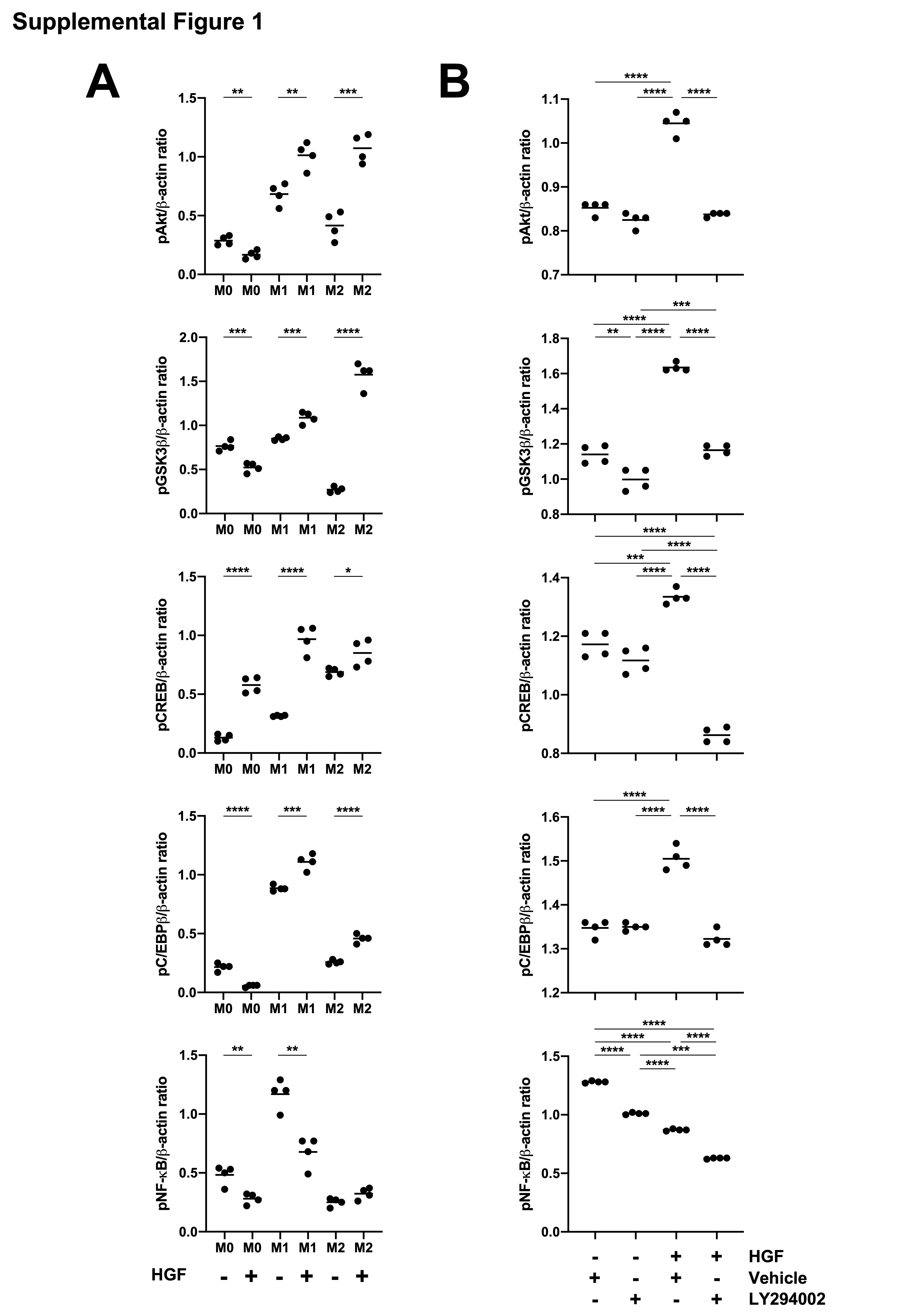

Supplement: Supplementary file 1 [file Image_1.JPEG]

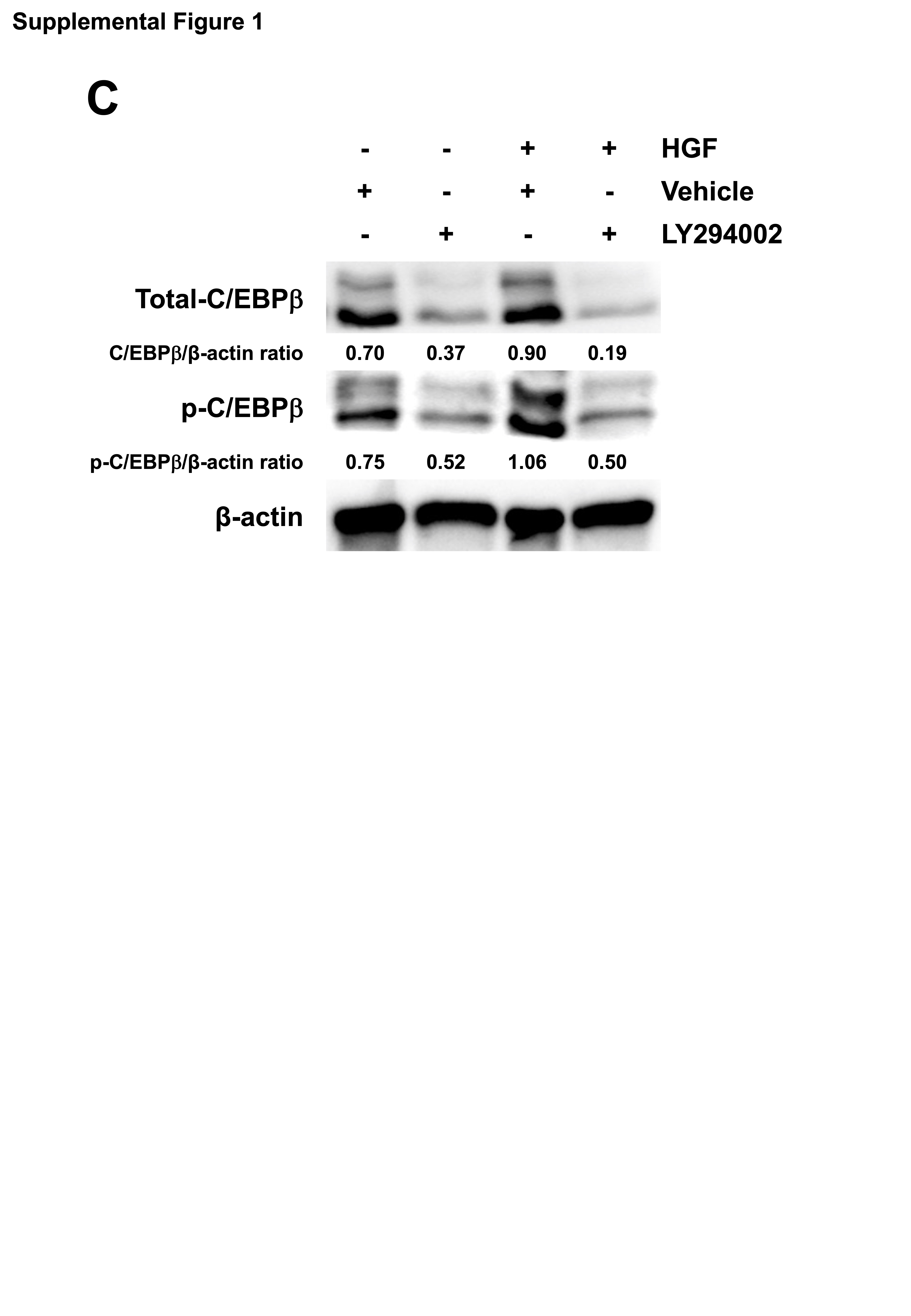

Supplement: Supplemental Figure 1 — (A,B) Quantification of Western blot bands based on Figures 3A,B. M1 macrophages were treated with a PI3K inhibitor (LY294002) for 1 h after PBS or HGF treatment for 24 h. (C) Effects of HGF treatment on phosphorylation of C/EBPβ in the presence and absence of a PI3K inhibitor. M1 macrophages were treated with a PI3K inhibitor (LY294002) with or without HGF for 24 h. Western blot bands were quantified by densitometry. The values were normalized to those of β-actin. The data are represented as the mean and a scatterplot showing individual data points (n = 4). Unpaired Student's t-test for (A), **P < 0.01, ***P < 0.001, and ****P < 0.0001. One-way ANOVA for (B), **P < 0.01, ***P < 0.001, and ****P < 0.0001. [file Image_2.JPEG]

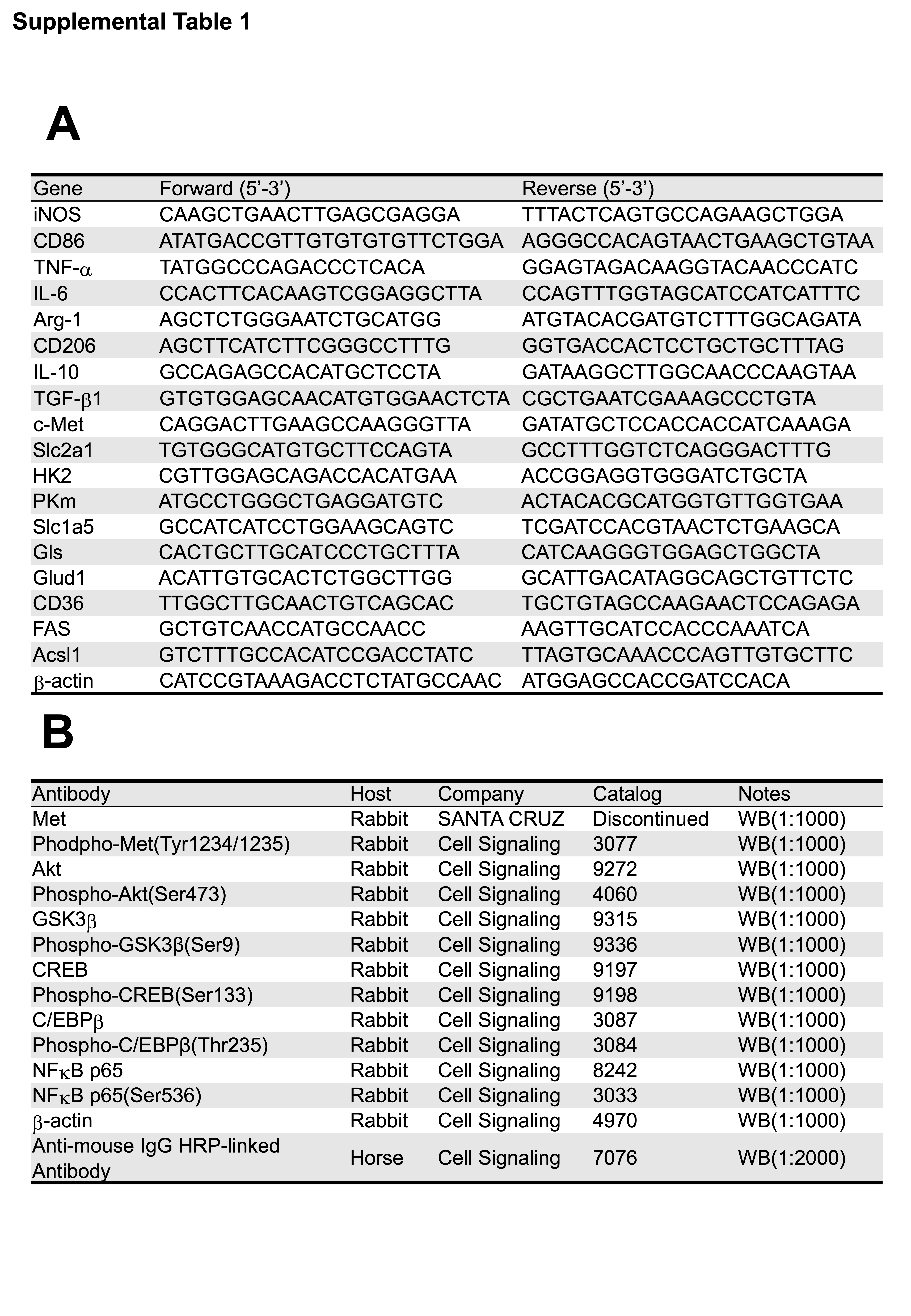

Supplement: Supplemental Table 1 — All primers and antibodies used in this study are listed. [file Image_3.JPEG]
